# The Role of Extracellular Vesicles in Embryo Implantation

**DOI:** 10.3389/fendo.2022.809596

**Published:** 2022-01-28

**Authors:** Kaiyu Chen, Junyong Liang, Ti Qin, Yunchao Zhang, Xi Chen, Zhengguang Wang

**Affiliations:** College of Animal Sciences, Zhejiang University, Hangzhou, China

**Keywords:** exosomes, microvesicles, embryo-maternal communication, embryo adhesion, micro-RNA (miRNA)

## Abstract

Extracellular vesicles (EVs) are membrane-coating nanoparticles derived from cells. The effect of cell-to-cell communication mediated by EVs has been investigated in different fields of physio-logical as well as pathological process in recent years. Reproduction, regarded as a definitive characteristic of organisms, has been a focus in both animal and medical sciences. It is well agreed that implantation is a critical event during early pregnancy in viviparous animals, and a proper implantation is essential for the establishment and maintenance of normal pregnancy. However, successful implantation requires the synchronized development of both the uterus and the embryo, therefore, in which well communication and opportune regulation are necessary. This review focuses on the progression of studies that reveal the role of EVs in early pregnancy, especially during implantation. Based on current evidence, EVs are produced and exist in the environment for implantation. It has been proved that EVs of different origins such as endometrium and embryo, have positive influences on embryo implantation. With their cargos of proteins and nucleic acids (especially microRNAs), EVs exert their effects including information transportation, immune stimulation and regulation of gene expression.

## Introduction

### Implantation and Its Requirement

Implantation is the key step of pregnancy establishment in mammalian reproduction, in which embryos are demanded to adhere to receptive endometrium and invade the latter. Successful implantation depends on the appropriate and coordinate state of both the maternal side and the embryo.

The embryo must develop into blastocyst stage with cells differentiated into the inner cell mass (ICM) and trophectoderm (TE). On the other side, the uterine epithelium must undergo characteristic changes into receptive state in response to estrogen(E) and progesterone(P), in which epithelial cells lose their polarity gradually and form microprotrusions on the apical surface (1, 2). Also, endometrium stromal cells (ESCs) proliferate and differentiate into decidual cells. When the uterus is well prepared, a short period, known as window of implantation, occurs for embryos to implant. Implantation out of window of implantation would lead to spontaneous miscarriages (3). Through the processes of apposition, attachment (adhesion) and penetration, the blastocyst implant to the receptive endometrium. During this period, communication between the two sides is essential for their synchronized development.

Implantation also requires a slightly inflammatory status in the uterus to facilitate tissue remodeling in spite of the necessity of tolerance for the embryo (4). In addition, it is conjectured that macrophages and dendritic cells help degrade the cover of endometrium for embryo implantation by producing cytokines and chemokines (4). However, the mechanisms of these processes are not yet clear.

Due to the complexity and difficulty of the process of implantation, the majority of conceptions fail and vanish actually. In fact, implantation failure causes most (75%) of the pregnancy losses in humans (5). This problem also distinctly restricts the pregnancy rates in assisted reproduction. Besides, benefit of animal breeding is affected by such a limit. In order to promote the reproduction of both humans and livestock, it is critical to understand the regulation mechanisms of embryo implantation.

### Extracellular Vesicles

**Figure 1:**
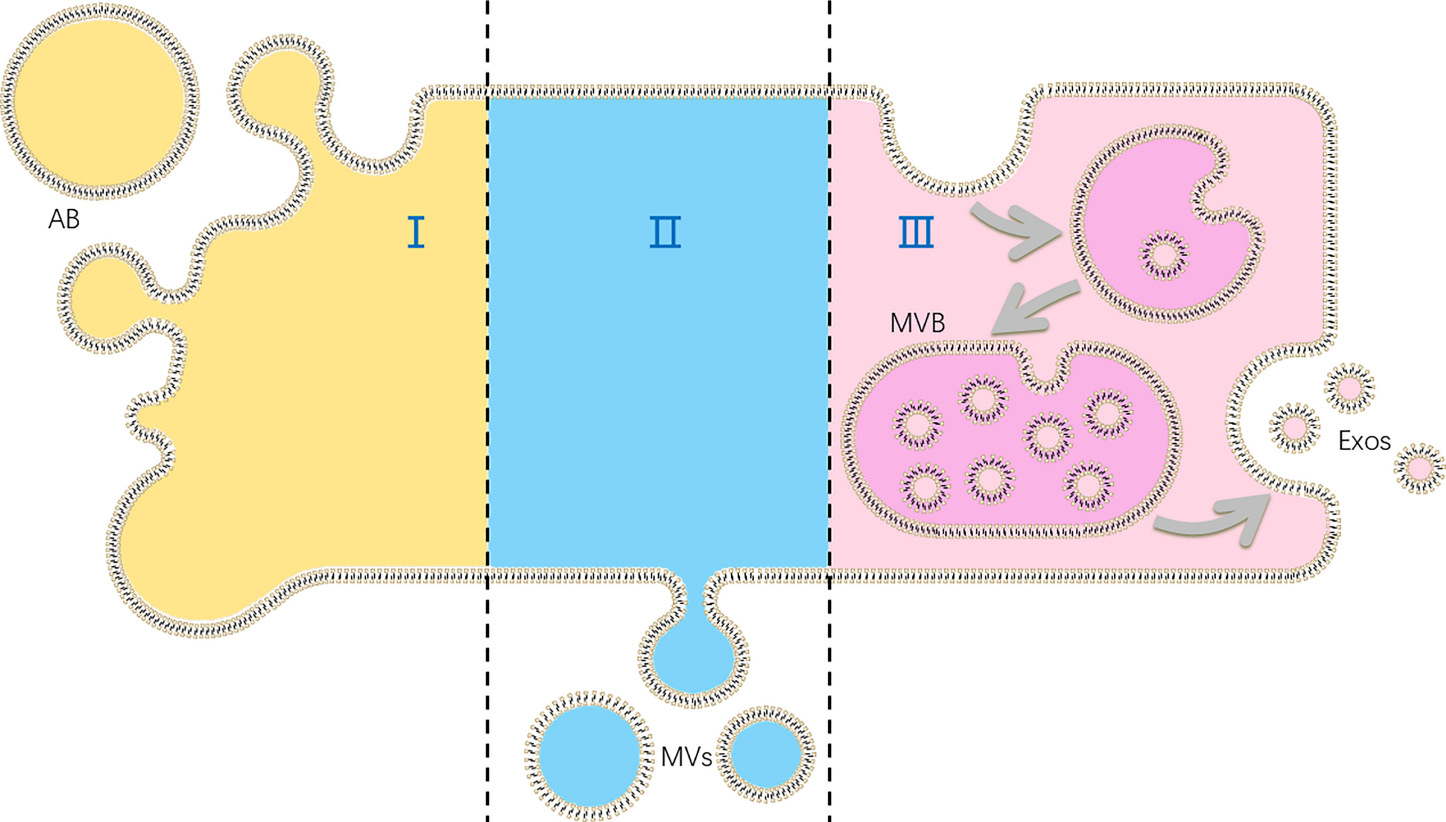
The production of different kinds of extracellular vesicles (EVs): (I) apoptotic bodies (ABs) are formed in cell apoptosis; (II) microvesicles (MVs) are shed directly by cells; (III) exosomes (Exos) are released as multi-vesicular body (MVB) fuse with plasma membranes.

Extracellular vesicles (EVs), as cell-derived membranous nano-sized vesicles, contain at least 3 main subgroups: apoptotic bodies, microvesicles and exosomes. Apoptotic bodies are released during apoptosis when plasma membrane blebbing, and are usually out of consideration of functions of EVs. Besides, microvesicles are produced by cell shedding directly, while exosomes originate from multi-vesicular body in cells, which form *via* two times of membrane bubbling (**Figure 1**). Although some authors suppose that EVs are produced by cells to discharge unnecessary molecules, the role of EVs as information transmitters is also demonstrated (6).

Different kind of EVs may act in different way. However, for the difficulty of fully separating microvesicles and exosomes, it is hard to distinguish functions of different kinds of EVs. Therefore, some of the studies refer EVs or small EVs (sEVs) instead.

EVs have been found to be released by most types of cells, and their capability of information transmission between cells provides a new mechanism of intercellular communication in addition to contact-dependent signaling and autocrine, paracrine, or endocrine signaling. Signals of EVs can be mediated by all different kinds of biomolecules including protein, lipids, nucleic acids and sugars, which can be delivered simultaneously and work together (7). Compared to directly secreted soluble molecules, signaling molecules within the membrane-derived EVs is protected from degradation of enzymes and their existence remains stable. Altogether, EVs play an important role in cell-to-cell cross-talk of many different physiological and pathological process (7).

The involvement EVs have been widely reported in vascular biology, immune responses, neurology, and reproduction (especially in embryo implantation) (7). Effects of EVs are reported more in oncology. Cancer-derived EVs are shown to promote the progress of cancer development and metastasis (8, 9). For the similarity between cancer development and embryo implantation that both go through epithelial-mesenchymal transition and change in cell capability of adhesion, migration and invasion, important role of EVs in embryo implantation can be reasonably assumed. In this review, we introduce findings about EVs during peri-implantation stage and try to summarize the role of EVs in the regulation of embryo implantation.

## Components and Origins of EVs During Peri-Implantation

EVs have been detected and isolated from tissues and fluid environments associated with embryo implantation during early pregnancy among species. Furthermore, physical and biochemical analysis of these EVs contribute to understanding of their classification, components and origins.

### EVs Exist in Uterine Flushing Fluids

The detection of EVs in uterine flushing fluids (UFs) have been reported in large farm animals such as sheep and cattle. A total of 195 proteins are identified in EVs from UFs of ewes, with 40 and 76 unique to the cyclic and pregnant ewes, respectively (10). Besides proteins such as cathepsin L1 and prostaglandin synthase two, UF EVs contain a large number of small RNAs including 81 conserved mature miRNAs, with 53 common in cyclic and pregnant ewes and 1 unique miRNA (bta-miR-423) in sample from pregnant ewes on day 14 (10). Among those, enJSRVs ENV and GAG RNAs, whose encoding proteins are shown to regulate the development of trophectoderm, are found to be delivered to other cells *in vitro* (10).

Exosomes are isolated from ovine UF in another study, with different protein cargos from day 15 and 17 cyclic (C15, C17) or pregnant (P15, P17) ewes (11). In this study, interferon tau (IFNT), macrophage-capping protein (CAPG) and aldo-keto reductase family 1, member B1 protein (AKR1B1) are found in exosomes isolated from P15 and P17 ewe UFs (11). IFNT contained in exosomes is believed to be involved in embryo implantation, while CAPG and AKR1B1 are also presumed to be relative to embryo attachment to the uterine epithelium.

In the subsequent study, total 596 proteins are detected in exosomes isolated from UFs of bovine on P17, P20 and P22. 172 differentially expressed proteins with more than 1.5-fold changes are found among these exosomes from different phases of pregnancy (12).

However, considering the complexity of the uterine cavity, EVs in UFs may have different origins such as seminal fluid, secretion of uterus and embryo, and even production of microorganisms.

### EVs Are Secreted by Uterus

Exosomes are isolated from immortalized ovine uterine glandular epithelial cells (13), indicating the capability of uterine epithelial cells to produce EVs. The surface markers of exosomes, CD9 and CD63, are detected on the apical surfaces of human endometrial epithelial cells (EECs), and CD63 shows cyclical regulation (14), indicating that exosomes are secreted by endometrial epithelium *in vivo* and are regulated during menstrual cycle. Exosome/microvesicle pellets are isolated and confirmed from culture medium of endometrial epithelial cell line (ECC1) and from uterine fluid. Profiling of miRNAs in exosomes/microvesicles derived from ECC1 shows that 214 miRNAs are present in both exosomes/microvesicles, while 13 miRNAs present specifically in exosomes/microvesicles and 5 are specific to cells (14), suggesting a sorting mechanism of miRNAs in secretion of exosomes/microvesicles from endometrial epithelium.

In another study, EVs are isolated from cultured women endometrial mesenchymal stromal cells (endMSCs), with their positive effects on blastomere division and embryo hatching of pre-implantation mice embryos and their potential for promoting angiogenesis demonstrated (15). The author also claims that in their preliminary proteomic analyses of the EVs derived from human endMSCs (EV-endMSCs), proteins related to embryo development and implantation are found, including transferrin, vinculin, and fibronectin for embryo development and matrix metalloproteinase-2, -3 and -9, and E-cadherin for embryo implantation (15).

Contents of endometrial epithelial-derived exosomes alter in the menstrual cycle or estrous cycle. A study demonstrates that different treatment of E and E+P could influence the proteome of exosomes derived from ECC1. Exosomes from the EP-treated ECC1, which represent the receptive phase of endometrial epithelium, contain 126 proteins that absent in E-exosomes (16). Furthermore, the protein cargo in EP-exosomes is believed to be related to implantation processes including adhesion, migration, invasion and extracellular matrix remodeling (16). The protein extracellular matrix metalloproteinase inducer (EMMPRIN) contained in microvesicles is secreted by human uterine epithelial cells (HESs), and is found to be regulated by ovarian hormones (17). Besides, 7 out of 768 miRNAs in EVs from uterine lumen of cyclic ewes are also regulated by progesterone (P4) (18). In the succeeding study, the production of EMMPRIN-containing microvesicles from uterine epithelium is found to be stimulated by GPR30 (19).

These studies show that uterus, especially endometrium, is capable for EVs secretion. The EVs production of endometrium alters along with the cyclic changes of uterus induced by E and P. Furthermore, the quantity and contents of EVs may reflect the characteristics of the physiological state of the uterus to a certain extent.

### Embryo Is Another Origin of EVs

EVs in uterine lumen during early pregnancy can also be of embryonic derivation. Evidence of exosomes-derived proteins presenting in both UF of pregnant heifers and culture medium of conceptuses, but are missing in the uterine tissue (20), indicates that the conceptus is capable of releasing selectively packaged EVs. EVs are demonstrated to be released by both uterus and day 14 conceptuses during early pregnancy in sheep, and these EVs are able to be transported to and internalized by the other side in the communication between conceptus and uterus (21). 512 mRNAs and 231 proteins are detected in the day 14 conceptus-derived EVs (21).

The study of exosomes in UF of ewes reveals that CAPG and AKR1B1 exist only in exosomes from UFs of pregnant ewes in peri-implantation period (P15 and P17), although exosomes are present in both cyclic and pregnant UFs (C15, C17, P15, and P17). Moreover, up-regulation of CAPG and AKR1B1 mRNAs as well as the expression of these two proteins are found to be specific to day 15 and 17 conceptuses compared to C15, C17, P15 and P17 endometrial tissues (11), indicating that exosomes are secreted to UFs by embryos during embryo attachment period.

After that, EVs are confirmed in human embryo spent medium, along with their contents of HLA-G protein and mRNAs for embryonic pluripotency-related genes including NANOG and POU5F1 (22). In consideration of the fact that NANOG is only expressed in the ICM but not in the TE at that time, it can be inferred that EVs are derived not only from the TE but also from the ICM (22). In another study, EVs and 621 miRNAs are found in *in vitro* fertilization (IVF) human embryo spent culture media, with different quantity between positive and negative outcome (6). Besides, microvesicles and exosomes are detected in culture media of bovine embryos on day 9 post IVF as well as parthenogenetic activation (PA), and non-competent IVF embryos show a higher variability in their EVs population (23). EVs are also found in mice outgrowth embryo-conditioned media by electron microscopy and Western blot in a study of peri-implantation embryos (24). These works again confirm that embryos can be source of EVs secretion.

DNA representing the full murine genome is also detected in EVs isolated from spent media of blastocyst (25). Interestingly, there is no difference in DNA cargos between apoptotic bodies and other EVs (25), which indicates the possibility of DNA cargos loaded and transported by microvesicles and exosomes during implantation.

EVs also exist in blastocoel fluid (BF), in addition to the external environment of embryos. EVs, mostly identified as exosomes, are discovered in human BF of pre-implantation embryos, along with 89 miRNAs (26), and most of which have been described as exosomal miRNAs. Pathway analysis and Gene Ontology analysis of these miRNAs reveals that cell stemness, cell reprogramming, cell communication, and cell adhesion are related processes (26). In an earlier study, microvesicles are isolated from conditioned medium (CM) of embryonic stem cells derived from the ICM (27), indicating that ICM could be origin of EVs in BF. These findings provide another possibility of EVs functioning in the development of pre-implantation embryos.

These researches reveal that embryos produce EVs during early pregnancy. Embryo-derived EVs can be originated from both ICM and TE. However, not all these EVs act on the mother side. EVs that exist in BF may contribute to cell-to-cell communication within the blastocyst.

### Other Origins of EVs in Female Reproductive Tract

Seminal plasma also contributes to EVs in uterus during early pregnancy. EVs in seminal plasma originate from the epididymal duct and the male accessory glands (7), and may remain in the uterus and act in the process of embryo implantation. It is demonstrated that EVs can be produced by helminths, parasitic protozoa and bacteria (7), and EVs from these lower organisms may exist in uterus. However, there is a lack of research about effects or functions of EVs from parasitic organisms in implantation.

## Effects of EVs During Peri-Implantation

**Figure 2:**
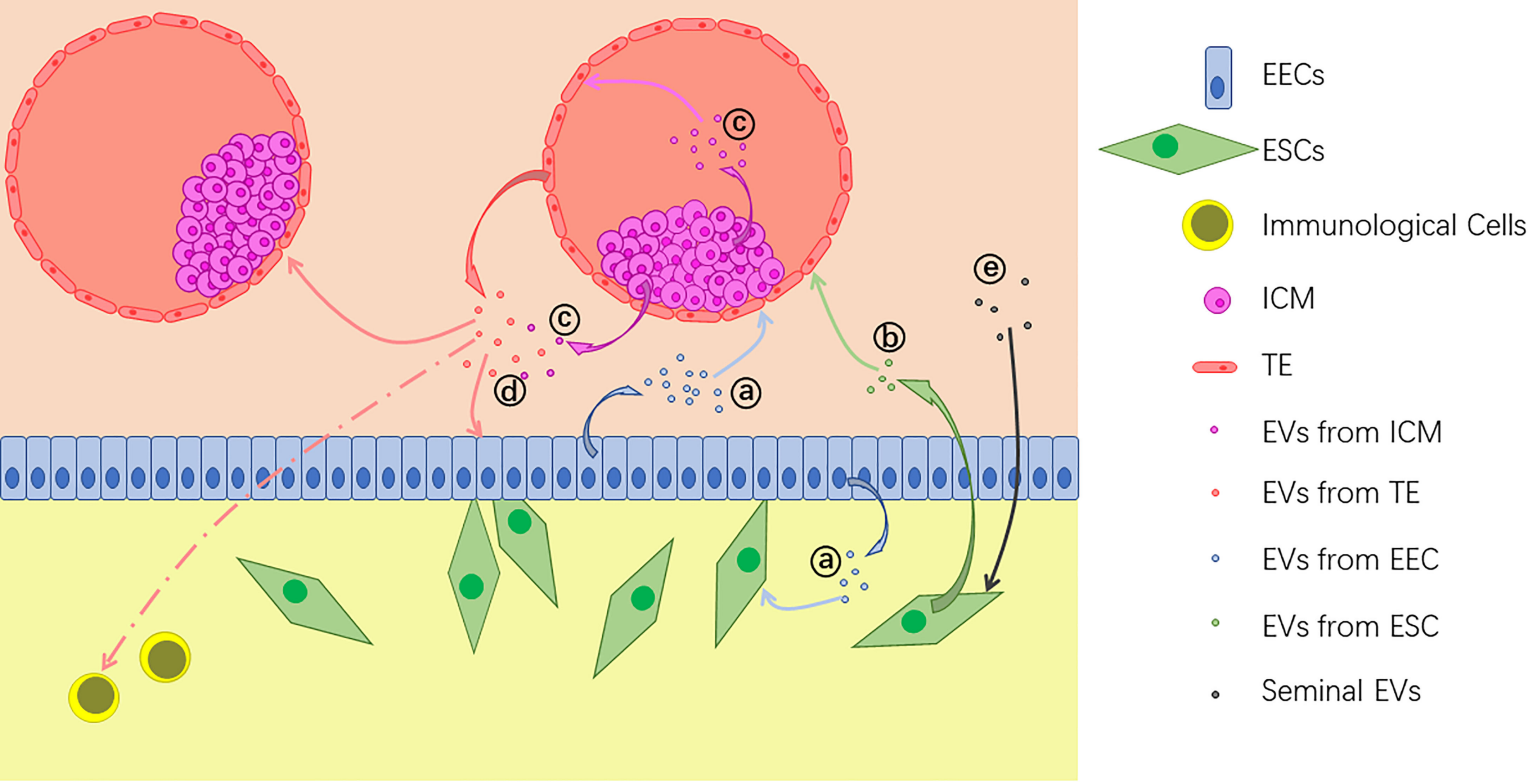
Extracellular vesicles (EVs) are released by different kinds of cells during implantation: **(A)** EVs from endometrial epithelial cells (EECs) act on the embryo and uterine fibroblasts; **(B)** EVs from endometrium stromal cells (ESCs) also act on the embryo; **(C)** EVs from the inner cell mass (ICM) act on the trophectoderm (TE) and some of them are transferred out of the blastocyst; **(D)** EVs from the embryo, including both TE and ICM, act on EECs, other embryos and immunological cells; **(E)** Seminal EVs act on ESCs.

It has been demonstrated that embryo-derived EVs can be taken up by both of epithelial and stromal cells in endometrium (22), and vice versa (15, 16). Besides, effects of EVs are also found in embryo-embryo (24, 28), ICM-TE (27), and other kinds of intercellular interactions. Also, effects of EVs alter along with the change of the origin. For example, exosomes from bovine UF on P17, which represents pre-implantation phase, are found to up-regulate the expression of apoptosis related genes in EECs. On the other hand, post-implantation exosomes of P20 and P22 up-regulate the expression of adhesion molecule VCAM1 (12). With their abundant and alterable components of proteins, nucleic acids and lipids, EVs can bring various changes to the target cells (**Figure 2**). In general, main effects of EVs during embryo implantation can be summarized as below.

### Promote Pre-Implantation Embryo Outgrowth and Development

It is suggested that embryos communicate with each other though EVs. Porcine PA embryos can improve cleavage and blastocyst formation rates of cloned (nuclear transfer, NT) embryos, and it is believed to be carried out by EVs containing specific mRNAs, which are internalized by NT embryos and increase the protein expression in the latter (29). Likewise, bovine NT embryos cultured in nonrenewal system show higher blastocyst quality compared with routine renewal treatment (28). Supplementation of exosomes isolated from culture medium to renewal system can significantly rescue blastocyst formation, quality and following growth (28). These studies of PA and NT embryos suggest that exosomes secreted by early embryo promote pre-implantation outgrowth and development of embryos and thereby advance their implantation capability.

Evidences of embryos from natural fertilization also prove that EVs derived from outgrowth embryos show embryotrophic effects to pre-implantation embryos in an incubation treatment. Supplementation of EVs to the culture medium of pre-implantation mice embryos significantly increases the average number of blastomeres in single blastocyst and at the same time, reduces apoptosis index in peri‐implantation embryos. Also, out-growth and trophoblastic spreading are increased and ICM grade is improved in peri-implantation embryos treated with EVs (24). These results demonstrate that EVs can improve development of pre-implantation embryos and help embryos prepare for implantation. More importantly, EVs-treated embryos show a significantly higher implantation rates in *in vivo* embryo transfer, which proves that EVs derived from outgrowth embryo-conditioned media indeed increase the implantation ability of mice embryos (24). Similarly, amniotic microvesicles show positive effect on bovine embryos hatching and subsequent pregnancy (30).

Embryos also benefit from EVs produced by the mother side. EVs derived from human endMSCs are found to significantly enhance total cell number of mice embryos when added to culture medium (15). Embryo hatching is also enhanced by EV-endMSCs compared to the non-EV-endMSCs added control (15), indicating that EV-endMSCs have positive influences on pre-implantation embryos towards implantation. In a recent study, similar results are also observed in the treatment of crude exosomes from hormone-treated EECs, in which human trophectodermal spheroids are used as blastocyst mimics (31). Embryo hatching depends on both mechanical and chemical processes. Embryo outgrowth and development contributes to the mechanical part through thinning zona pellucida, which is proved to be promoted by EVs. However, it remains unknown if EVs participate in the chemical part.

### Improve Cell Migration, Adhesion and Invasion

In a study of exosomes isolated from plasma of pregnant women shows that placental exosomes can promote endothelial cell migration (32), suggesting that cell migration and invasion may also be enhanced by EVs during implantation.

Endometrial exosomes perform different influence in HTR8 trophoblast cells according to hormonal treatment to their original ECC1. In comparison to exosomes derived from E-treated ECC1, EP-exosomes rapidly and significantly increase the adhesive capacity of HTR8 cells after internalization, which is in accordance with the result of their proteomic analysis (16).

The subsequent study finds that spheroids of trophectodermal cells are influenced by ECC1-derived EVs in response to EP treatment and represent significantly promoted outgrowth on fibronectin and more importantly, increased adhesion and invasion. The enhanced adhesion can be partly reduced by EV uptake inhibitors, testifying the effect of endometrial EVs (33). It can be inferred from this research that adhesion and invasion of blastocysts, represented by spheroids of trophectodermal cells in the study, can be positively regulated by endometrial EVs, and therefore implantation is promoted. Another study also demonstrates that sEVs derived from human decidual stromal cells (HDSCs) enhance cell invasiveness of trophoblast after the uptake by the latter (34).

Microvesicles generated by embryonic stem cells derived from ICM can be transferred to the trophectoderm and enhance the outgrowth and migration of trophoblast (27). More importantly, injection of embryonic stem cell microvesicles to blastocysts significantly enhance their ability of implantation (27), indicating that microvesicles mediate the intercellular communication in blastocysts and promote the process of implantation.

### Other Effects Contributing to Implantation

Molecules including vascular endothelial growth factor (VEGF) and platelet-derived growth factor-AA secreted from EV-endMSCs-treated embryos shows potential stimulation effect of EV-endMSCs on feedback signal of embryos, which help to prepare uterus for implantation and following processes (15). This research indicates that EVs may perform their influence during peri-implantation through the regulation of cell secretion.

IFNT, as an important pregnancy recognition factor of ruminants, is detected in exosomes from UF of pregnant ewes but not cyclic ones (35), indicating that IFNT is secreted by conceptus trophectoderm. Conceptus trophectoderm cells (oTr1) treated with exosomes isolated from UF of ewes on day 13 of the estrous cycle are found to gain higher rates of proliferation and increased production of IFNT (35). This study presents an instance of EVs acting as mediator in communication between conceptus and uterus in pregnancy recognition.

Seminal exosomes can be internalized by human ESCs and induce the production of cytokines relevant to implantation, IL6 and IL8, of the latter (36). In another study, microvesicles from seminal plasma are proposed to promote decidualization of human primary endometrial stromal fibroblasts (ESFs) (37), which is essential to the process of human embryo implantation. Also, IL11 production of ESFs is induced by seminal microvesicles (37). These two studies suggest a role of seminal EVs in the regulation of implantation by seminal plasma in both immunology and cell differentiation.

Effect of trophoblast-derived EVs is also found on monocytes. Exosomes derived from Sw.71 trophoblast cells are rapidly taken up by monocytes, and the migration of the latter is promoted in a chemotactic way. Besides, the expression and secretion of cytokines related to early pregnancy in monocytes and macrophages are induced by exosomes (38). These effects can contribute to embryo implantation by supporting pro-inflammatory reaction, angiogenesis and stroma remodeling.

Interestingly, EVs derived from defective origins seem to act in an opposing way, or at least in an ineffective way. Exosomes from UFs of cows with endometritis represent a negative effect on development of IVF embryos compared to the healthy ones (39). Treatment with EVs from endometrial cells of women with recurrent implantation failure also reduces the hatching rates and total cell number of blastocysts (40). Furthermore, the capability of invasion of hatched blastocysts is decreased with treatment of such EVs compared to normal ones (40). These results suggest that effects of EVs may be associated with health condition of the subject.

## Possible Mechanisms of EVs Functioning in Embryo Implantation

The detail of how EVs exert their functions is not yet clear, especially considering the complicacy of the components of EVs and the target cells. However, there have been many studies making efforts into this field, therefore we could get an insight into which.

For example, the mechanism of EVs altering cell capability of adhesion and migration is studied. ECC1-derived exosomes are found to significantly increase the expression level of adhesion markers including focal adhesion kinase (FAK), phosphorylated FAK (Tyr397), and fibronectin in trophoblast cells (16). Also, the expression of FAK and FAK-Tyr397 is induced to a significant higher level by EP-exosomes compared to E-exosomes (16). This research suggests that endometrial exosomes enhance adhesive capacity of trophoblast cells through activating FAK signaling (16). Another study points out that HDSC-sEVs upregulate the N-cadherin expression by inducing the phosphorylation of SMAD2 and SMAD3, and therefore, improve trophoblast cells migration and invasion (34).

However, effects of EVs on target cells are multiple and therefore mechanisms would be complicated. Protein expression of human trophectodermal spheroids is attested to be reprogrammed by ECC1-derived EVs. Biological processes of many up-regulated proteins are associated with implantation, including actin cytoskeleton regulation, cell adhesion and cell division, while extracellular matrix organization and cell adhesion were down-regulated functions (33). Implantation relative proteins whose expression are modulated by EVs are identified in trophectodermal spheroids (33), including S100A10, EPCAM, and PFN2. Several proteins secreted by trophectodermal spheroids are up-regulated uniquely in response to EVs, including chemokine (C-X-C motif) ligand 12 (CXC12), CXCL8, alkaline phosphatase, cysteine-rich protein 61, tissue factor inhibitor, and integrin alpha 6. These proteins are found related to cell and substrate adhesion, cell migration, and binding capacity (33), suggesting a feedback signaling of blastocyst in response to endometrial EVs in pre-implantation maternal-embryo communication.

According to existing studies, these functions of EVs during peri-implantation stage are mainly carried out by two kinds of molecular components, proteins and nucleic acids.

### Protein Cargos React With Target Cells

A large number of functions of EVs during peri-implantation stage are carried out by protein cargos. EV-mediated signals can be delivered and transduced by interactions between EV proteins and target cells and the target cells make response through downstream reactions. For example, as mentioned above, many kinds of proteins related to embryo development and implantation are detected in EV-endMSCs (15). It can be conjectured that proteins delivered by EVs can react with their target cells to promote embryo blastomere division and hatching and afterwards, embryo implantation. More clear evidences are below (**Table 1**).

**Table 1:** EV Proteins and their functions in embryo implantation.

Proteins	Functions	Origin	Kind of EVs
enJSRVs (35)	initiate synthesis and secretion of IFNT in conceptus trophectoderm cells	uterus (sheep)	exosomes
IFNT (35)	regulate expressions of conceptus attachment-related genes	embryo (sheep)	exosomes
CAPG (35)	enhance cellular motility and chemotaxis	embryo (sheep)	exosomes
AKRB1 (35)	regulate epithelial-mesenchymal transition and angiogenesis	embryo (sheep)	exosomes
INFT (12)	regulate expressions of conceptus attachment-related genes	UFs (cattle)	exosomes
CD40L (41)	induce the expression of pro-inflammatory genes in EECs	UFs (cattle)	exosomes mainly
EMMPRIN (17)	stimulate the MMP expression in HUFs	HES cells (human)	microvesicles
IL11 (37)	promote decidualization of ESFs	seminal plasma (human)	microvesicles
Fibronectin (42)	induce IL1β production of macrophages	trophoblast (human)	exosomes
Laminin and fibronectin (27)	promote trophoblast migration	embryonic stem cell (mouse)	microvesicles
Phosphatidylserine (43)	improve IL10 production of T cells	embryo (mouse)	(not memtioned)

Endometrial remodeling is proved to be regulated by EV proteins. EMMPRIN is known as a regulator of uterine fibroblast production of matrix metalloproteinases (MMPs), which direct endometrial remodeling during implantation. The EMMPRIN protein is found to be secreted by HES cells through microvesicles and stimulate the MMP expression in human uterine fibroblasts (HUFs) (17).

Proteins in EVs participate in both direction of communication for pregnancy recognition in ruminants. A study proposes that toll-like receptors (TLRs) on conceptus trophectoderm cells, especially TLR7 and TLR8, can recognize exosomal enJSRVs from uterus and initiate cell signaling resulting in synthesis and secretion of IFNT (35). On the other side, IFNT, as well as CAPG and AKR1B1 are contained in embryo-derived exosomes isolated from P15 and P17 ewe UFs (11). Interferon stimulated genes (ISGs) mRNA expressions in EECs are up-regulated after treated with these exosomes, which is believed to be carried out by IFNT identified in them (11). IFNT has been proved to regulate many endometrial gene expressions of proteins which are critical for conceptus attachment to endometrial epithelium, including CXCL10 and galactoside-binding, soluble, 15. Besides, CAPG can enhance cellular motility and chemotaxis, and is involved in increased cell invasion. Moreover, AKR1B1 is also known as a molecule related to epithelial-mesenchymal transition and angiogenesis. Embryo-derived exosomes may also promote implantation through these two proteins, though more evidences are required.

In the following study, exosomes isolated from bovine UFs are again found to alter the gene expression of EECs. The transcriptions of ISG15, myxovirus resistance (MX)1, MX2, STAT1 and STAT2 are up-regulated by exosomes from P17, P20 and P22 UFs in an IFNT concentration dependent manner (12). Thus, these up-regulations of gene expression is believed to be result from IFNT in exosomes.

IFNT-independent effects of EVs in embryo implantation of cattle are also found in a recent study (41). RNA-seq analysis of bovine EECs reveals 82 transcripts induced by IFNT-independent P17 EVs, many of which are associated with the TNF pathway and inflammatory response (41). A higher expression level of CD40 ligand (CD40L) is found in P17 EVs compared to C17 ones. P17 EVs can induce the expression of TNF signaling pathway-related genes in EECs *via* CD40L-CD40-NF-κB pathway, independent of IFNT effects, to provide a pro-inflammatory environment for implantation (41).

The positive effects of embryonic stem cell microvesicles on the capability of migration of trophoblast are proved to be mediated by laminin and fibronectin together. These two proteins on the outer surfaces of microvesicles can bind to integrin α5β1 and the laminin receptor on trophoblasts, which activates FAK and c-Jun N-terminal kinase pathways and thereby trophoblast migration is promoted (27).

EVs from seminal plasma exert their effects on embryo implantation at least partly with protein cargos. Seminal microvesicles promote decidualization of ESFs with proteinaceous components, and this effect is found to be IL11 signaling-dependent (37).

EVs proteins also act on immunological cell during implantation, for that slight inflammation is required in endometrium to allow the process of implantation and trophoblast invasion (4). The chemotactic migration of monocytes and macrophages and their production of cytokines including IL1β are advanced by trophoblast-derived exosomes and lead to a pro-inflammatory environment in uterus (38). A following research demonstrates that exosomal fibronectin is responsible for inducing the expression and release of IL1β by human macrophages without exosome internalization (42). This stimulating function of fibronectin is found to be mediated by specific cell surface α5β1 integrin receptor.

The adhesion of EVs derived from mouse embryo to CD4+ and CD8+ murine peripheral T lymphocytes is found to be partly mediated by phosphatidylserine- phosphatidylserine receptor binding (43). Also, IL10 production of CD8+ T cells is improved by EVs *via* their protein cargo of progesterone-induced blocking factor (43), which is known as a protein mediating the immunological actions of progesterone and therefore participates in the embryo-maternal communication.

### Nucleic Acid Cargos Influence Gene Expression of Target Cells

Nucleic acid cargos, especially microRNAs have been the hotspot and focus of researches about EVs. The important role of miRNAs in embryo implantation has been summarized in earlier reviews, including one of our group (44, 45). MiRNAs in EVs are believed to be involved in regulation of uterine gene expression (44). For example, bioinformatics analysis of miRNAs in exosomes/microvesicles derived from ECC1 shows that a number of target genes of these miRNAs are known as relative to implantation (14), which include adherens junctions, ECM-receptor interactions, the VEGF-signaling pathway, the Jak-STAT pathway and the Toll-like receptor signaling pathway.There have been more evidences of nucleic acid and especially miRNAs in EVs functioning during peri-implantation.

RNAs are proved to be transferred from embryo to endometrium through EVs. 3 unique and abundant RNA fragments are found to significantly down-regulate the expression of their original genes. One of these RNA fragments originates from ZNF81 gene, and the others are from LINC00478 locus of chromosome 21, which transcripts matches with LTR7B family and is close to a region potential for endogenous retrovirus protein (46). Notably, LTR7 copies are involved in the regulatory network of pluripotency in human embryonic stem cells, while endogenous retrovirus proteins are necessary for early embryo and placenta development during implantation. Interestingly, EVs derived from good-prognosis IVF embryos show the potential to alter ZNF81 gene expression in endometrial cells, while EVs from poor prognosis embryos are not able to initiate such changes (46). Therefore, we can reason that RNA cargos in embryo-derived EVs may act as markers of embryo quality and be recognized by the mother side.

There are more evidences about EV RNAs influencing gene expression in cattle EEC. Transcriptome analysis reveals that there are 179 differentially expressed genes between EECs co-cultured with EVs isolated respectively on P17 and P20. Among these differentially expressed genes, Gene Ontology and pathway analyses reveal that RNAs related to immune response and immune system are down-regulated in EVs from P20 (47). Furthermore, bta-miR-98, which is more abundant in P20 EVs, is found to down-regulate immune system-related genes in EECs. These results indicate that miRNA in EVs are involved in the regulation of maternal immune system during the peri-implantation period to allow embryo implantation (47).

On the other side, miRNAs from EEC-derived EVs influence embryos in mouse and human. Our group proves that miR-100-5p enriched in receptive endometrium cell-derived EVs promotes cell migration, invasion, and proliferation of embryos and enhances their ability for implantation (48).

It is suggested that the differently expressed miRNAs in exosomes is the key to different effects on development of IVF embryos between healthy cows and those with endometritis (39). A total of 118 miRNAs are found to be expressed differently in exosomes of the two group, the author also suggests 20 of which as markers of early endometritis (39).

Our group recently proposes another possible pattern of EVs functioning during implantation with miRNAs cargos. Compared to the pre-implantation period, content of miR-34c-5p, miR-210, miR-369-5p, miR-30b, and miR-582-5p in endometrium-derived sEVs is remarkably increased during window of implantation (49). 2987 mRNAs as potential targets of these miRNAs are mainly enriched in the phosphatidylinositol 3 kinase/protein kinase B signaling pathway, mitogen-activated protein kinase signaling pathway, focal adhesion, cell–cell adhesion, and extracellular exosome (49). In addition, there is a remarkably higher expression level of miR-34c-5p, which is then proved to trigger embryo loss, in HEC-1-A cells, which represent non-receptive endometrium, compared to Ishikawa cells. However, GAS1, which is found to be negatively regulated by miR-34c-5p, is expressed at a higher level in Ishikawa cells, as a model of receptive endometrial cells (49). Therefore, miR-34c-5p in endometrium-derived sEVs may be castoff of endometrial cells in the conversion into receptive state, and endometrial cells may unload certain miRNAs by releasing EVs to prepare for embryo implantation. However, more effects of miR-34c-5p in sEVs are not yet well understood.

## Conclusions and Outlooks

It has been demonstrated with all these evidences that EVs play an important role in the communication and regulation in embryo implantation. Both of microvesicles and exosomes are secreted during peri-implantation period, and their origins are extensive, including endMSCs, HDSCs, EECs, as well as embryonic cells of TE and ICM. EVs mediate the bidirectional communication between maternal side and embryos to promote the coordinate changes. Besides, EVs also act as immune regulators to ensure an appropriate pro-inflammatory environment for implantation. Thereinto, proteins and nucleic cargos of EVs, for example, IFNs and miRNAs, are the keys that cause series of reactions in target cells to benefit the process of implantation.

However, there still remain a number of questions. For instance, how is the sorting of molecules regulated before EVs release? Can the amount or contents of EVs act as marker of stage and condition of embryo or uterus? In all these fields, more details and *in vivo* evidences of functions and involved pathways of EVs also await further study. For example, in another study of our team, miR-133a is found in exosomes from endometrial cancer cells (Ishikawa and HEC-1-A) (50). More importantly, FOXL2, a down-regulated key gene in endometrial cancer, is regulated by miR-34c-5p (50). Also, these exosomes could be transferred to normal endometrial cells (50), but more studies about their effects and mechanisms are needed. Also, endometrial EVs, especially exosomes, are proposed to represent gene expression variability at different time in menstrual cycle (51). However, there is still limitation of sample size and lack of sample from healthy women in this study (51).

Taking advantage of our understanding of EVs during implantation, new technologies can be developed to promote the reproduction of both human and livestock. For example, human EV-endMSCs can partly rescue embryo yield and quality in aged mice, and increased odds of implantation and subsequent delivery can be reasonably expected (52). Besides, artificial EVs can also be hoped to deliver specific molecules including medicines in order to encourage or break the process of implantation. Also, understanding and then regulating the endogenous release of EVs may achieve the same purpose.

In conclusion, there are still much to be explore in this field, and more efforts are demanded in succeeding works.

## Author Contributions

Collection of published articles: KC and YZ, Writing—original draft preparation: KC and JL. Writing—review and editing: TQ, XC, and ZW. Funding acquisition, ZW. All authors have read and agreed to the published version of the manuscript.

## Funding

This research was supported by grants from the National Natural Science Foundation of China (32172724), Department of Science and Technology of Huzhou City (2019ZD2026), the Sanya Yazhou Bay Science and Technology City 484 (202002007), and Zhejiang Team Science and Technology Commissioner Project 485 (Tongxiang).

## Conflict of Interest

The authors declare that the research was conducted in the absence of any commercial or financial relationships that could be construed as a potential conflict of interest.

## Publisher’s Note

All claims expressed in this article are solely those of the authors and do not necessarily represent those of their affiliated organizations, or those of the publisher, the editors and the reviewers. Any product that may be evaluated in this article, or claim that may be made by its manufacturer, is not guaranteed or endorsed by the publisher.
